# Gas stunning with CO_2_ affected meat color, lipid peroxidation, oxidative stress, and gene expression of mitogen-activated protein kinases, glutathione S-transferases, and Cu/Zn-superoxide dismutase in the skeletal muscles of broilers

**DOI:** 10.1186/s40104-018-0252-2

**Published:** 2018-05-09

**Authors:** Lei Xu, Haijun Zhang, Hongyuan Yue, Shugeng Wu, Haiming Yang, Zhiyue Wang, Guanghai Qi

**Affiliations:** 1 Key Laboratory of Feed Biotechnology of Ministry of Agriculture & National, Engineering Research Center of Biological Feed, Feed Research Institute, Chinese Academy of Agricultural Sciences, No. 12 Zhongguanchun South Street, Haidian District, Beijing, 100081 China; 2 College of Animal Science and Technology, Yangzhou University, No. 48 Wenhui East Road, Yangzhou, 225009 Jiangsu China

**Keywords:** Antioxidant enzyme, Broiler, Controlled atmosphere stunning, Gas stunning, Lipid peroxidation, MAPK/Nrf2/ARE, Meat color, Oxidative stress

## Abstract

**Background:**

Meat color and lipid peroxidation are important traits related to meat quality. CO_2_ concentration is a critical factor that can affect meat quality in the commercial use of gas stunning (GS). However, the effect and mechanism of CO_2_ stunning on meat color and lipid peroxidation during long-term storage remain poorly studied. We aimed to study the effects of GS methods, especially CO_2_ concentration, on meat color and meat lipid peroxidation in broilers during long-term storage at 4 °C and to explore the potential mechanism of meat color change via lipid peroxidation and the inner lipid peroxide scavenging system.

**Methods:**

Eighteen broilers were sacrificed after exposure to one of the following gas mixtures for 90 s: 40% CO_2_ + 21% O_2_ + 39% N_2_ (G40%), 79% CO_2_ + 21% O_2_ (G79%), or no stunning (0% CO_2_, control). Meat color, serum variables, enzyme activities, and the gene expression of mitogen-activated protein kinase (*MAPK*), nuclear factor-erythroid 2-related factor 2 (*Nrf2*), glutathione S-transferase (*GST*) and superoxide dismutase (*SOD*) were determined.

**Results:**

The concentrations of serum triiodothyronine (T3, *P* = 0.03) and the ratio of serum free triiodothyronine/free thyroxine (FT3/FT4, *P* <  0.01) were decreased, whereas levels of serum cortisol (*P* <  0.01) were increased in the 40% CO_2_ group compared with the control group. Additionally, the thiobarbituric acid-reactive substances (TBARS) _3 d_ (*P* <  0.01) and TBARS _6 d_ (*P* = 0.01) in breast meat and the TBARS _3 d_ in thigh meat (*P* <  0.01) were increased in the 40% CO_2_ group compared with the control group. Serum T3 was negatively correlated with TBARS_6 d_ both in the breast and thigh meat (*r* = − 0.63, *P* <  0.01 and *r* = − 0.47, *P* = 0.05 respectively). T3/T4 was negatively correlated with TBARS_6 d_ in the breast meat and in the thigh meat (*r* = − 0.57, *P* = 0.01; and *r* = − 0.53, *P* = 0.03 respectively). Compared with the control group, Lightness (L*) _1 d_ (*P =* 0.03) and L*_9 d_ (*P <* 0.01) were increased, whereas total chromatic aberration (E*) _1 d_ (*P =* 0.05) and E*_3 d_ (*P <* 0.01) were decreased in the breast meat of both the G40% and G79% groups. The values of yellowness (b*) _3 d_ (*P =* 0.01), b*_6 d_ (*P <* 0.01) and E*_6 d_ (*P <* 0.01) in the thigh meat were lower in both the G40% and G79% groups than in the control group. In the breast muscle, the mRNA levels of c-Jun N-terminal kinase 2 (*JNK2, P* = 0.03), *GSTT1* (*P* = 0.04), and *SOD1* (*P* = 0.05) were decreased, and the mRNA levels of *JNK1* (*P* = 0.07), *Nrf2* (*P* = 0.09), and *GSTA3* (*P* = 0.06) were slightly lower in both the G40% and G79% groups compared with the control group. However, among these genes, only the mRNA level of *JNK1* was decreased in the G40% group compared with the control group and the G79% group (*P* = 0.03) in the thigh muscle.

**Conclusions:**

Compared with the control group, meat color quality in the breast meat was decreased, and the expression of genes in the MAPK/Nrf2/ARE (antioxidant responsive element) antioxidant pathway in breast muscle was partly suppressed by GS of both 40% and 79% CO_2_. However, oxidative stress and meat lipid peroxidation during storage were aggravated by GS with 40% CO_2_ compared to GS with 79% CO_2_ and no GS.

## Background

To maintain animal welfare and meat quality in the preslaughter stunning system, gas stunning (GS, also called controlled atmosphere stunning, CAS) is considered the most promising alternative method of electrical stunning (ES). GS is replacing traditional ES in large-scale slaughterhouses [1] in many countries, especially in areas in which the legislation on animal welfare is strict, such as Europe [2]. The effect of GS on meat quality has been described in numerous publications. These meat quality traits mainly included meat tenderness, water holding capacity, pH, and carcass defects. Meat color is an important quality in the consumer’s evaluation and acceptance of a meat product at the time of consumption [3]. Previous studies of the effects of GS on meat color used a variety of parameters. These studies were mainly focused on the comparison of different stunning methods, and the study of meat color was limited to within 24 h postmortem [4–7]. The effects of GS on the meat color of poultry during long-term storage were not studied. Natural air is composed of approximately 21% O_2_, 78% N_2_ and 1% other gases. Two options exist to induce coma by commercial GS (or CAS) methods. One is by reducing O_2_ in the gas to induce hypoxia (as in the low atmospheric pressure stunning-killing system), and the other is by enhancing the CO_2_ concentration in gas to induce hypercapnia. In previous studies of the effects of GS on meat color, either both CO_2_ and O_2_ concentrations were enhanced [4], CO_2_ was enhanced and O_2_ was decreased simultaneously [6], or O_2_ was decreased (low atmospheric pressure) [7]. However, the effect of CO_2_ on meat color was rarely studied. In our previous study [5], O_2_ was fixed at the level in the air (21%) and the effect of CO_2_ (40 and 60%) on meat color within 24 h postmortem was studied. In the present study, we studied the effect of CO_2_ concentration on meat color after a prolonged storage time at 4 °C with a normal O_2_ concentration (21%) and a wider range of CO_2_ concentrations (40% vs. 79%).

Meat discoloration can be caused by myoglobin oxidation and lipid oxidation [8]. Additionally, by-products generated by lipid oxidation have cytotoxic and genotoxic properties and the higher consumption of heavily oxidized meat can be a great threat to human health [9]. Meat oxidation can also reduce shelf life [10]. The relationship between preslaughtering factors and lipid peroxidation needs to be clarified [11]. However, the effects of CO_2_ stunning on the lipid peroxidation of poultry, especially during long-term storage, have rarely been reported. In our previous study, lipid peroxidation within 24 h postmortem was observed to be affected by different stunning methods (GS vs. ES). However, this difference was not detectable between different CO_2_ concentration (40 and 60%) groups, or between CO_2_ stunning groups and the control group [12]. The concentration used in the previous study was moderate. In the current study, a wider range of CO_2_ concentration (40% vs. 79%) was used to study the effect of CO_2_ stunning on lipid oxidation during prolonged storage time at 4 °C.

The mechanism behind the effects of CO_2_ stunning on meat lipid oxidation was also not clear. One factor could be the residual haemoglobin content in the meat. A higher haemoglobin content in the CO_2_^-^stunned (but not bled) birds resulted in higher thiobarbituric acid-reactive substances (TBARS) values in the raw meat of broilers compared with CO_2_^-^stunned and bled birds [10]. Additional mechanisms behind the effects of CO_2_ stunning on lipid peroxidation need to be explored. In general, the mechanism of lipid peroxidation is free radical chain reaction, and reactive oxygen species (ROS) and iron (such as “free” iron in myoglobin) are the primary initiators and major catalyst, respectively [11]. The low “storage-stable” ferric ion reducing capacity in chicken breast was responsible for the high oxidative stability of raw chicken breast under storage conditions [11]. Oxymyoglobin oxidation is a process that is affected mainly by two pathways: the first one generates active oxygen species such as O_2_•^−^ and H_2_O_2_, and the second one generates lipid peroxides and lipid free radicals [13]. Thus, meat lipid peroxidation can be regulated by mediating ROS production or the muscle’s lipid peroxide scavenging system.

Our previous results showed that lipid oxidation and ROS were affected by GS methods and were reduced by the up-regulation of avian uncoupling protein and avian peroxisome proliferator-activated receptor-γ coactivator-1α expression in skeletal muscles at 45 min postmortem; however, meat lipid oxidation was not affected at 24 h postmortem [12]. Meat lipid peroxidation can also be regulated by the muscle inner peroxide scavenging system, including non-enzymes, antioxidant enzymes, and phase II enzymes. Therefore, the current study focused on the phase II enzyme system and long-term meat storage. The expression levels of phase II enzymes, such as glutathione S-transferase (GST), heme oxygenase-1 (HO-1), and nicotinamide adenine dinucleotide phosphate (NADPH): quinone oxidoreductase 1 (NQO1), are under the control of nuclear factor-erythroid 2-related factor 2 (Nrf2, or chicken erythroid-derived CNC-homology factor, ECH) / antioxidant responsive element (ARE) signaling [14–16]. Modulation of the Nrf2/ ARE axis through mitogen-activated protein kinase (MAPK) signaling [16] leads to the expression of phase II antioxidant enzyme in various cell types [17, 18] and adaptation to various extracellular stresses [19]. Muscle antioxidant capacity and meat quality were reported to be improved and exhibited changes in Nrf2-related signaling molecule expression, as a result of long-term factors during the survival period of animals, such as the level of dietary arginine [20]. In our previous study, under the condition of very acute (approximately 18 s) electrical stunning stress, genes in the MAPK/Nrf2/ARE pathway were transcriptionally activated in breast muscle by low-current and high-frequency electrical stunning [21]. However, the potential effect of short-term GS stress (approximately 1.5 min) on lipid peroxidation, meat color, and the MAPK/Nrf2/ARE antioxidant signaling pathway postmortem was not clear.

Thus, in the current study, we examined the effects of GS with different CO_2_ concentrations on meat color and lipid peroxidation in the meat of broilers during long-term storage at 4 °C, and we explored the potential involvement of the inner lipid peroxide scavenging system via the analysis of serum variables, TBARS, phase II enzymes, and gene expression in the MAPK/Nrf2/ARE signaling pathway in the skeletal muscles.

## Methods

### Animals and housing

Day-old Arbor Acres broilers (males, *n* = 60) were reared under the same environment for 49 d in the chicken facility at our institute. Water and the same diet were provided ad libitum until capture at 20 min before stunning on the 49^th^ day.

### Experimental design

Among the commercially used GS methods, CO_2_ and O_2_ concentrations are the most relevant factors that affect stunning efficiency and meat quality. However, N_2_ is considered inert for poultry. Thus, in the present study, the O_2_ concentration was standardized among the treatment groups, leaving CO_2_ concentration as the only variable. Before stunning, 18 birds weighing 2.50 ± 0.25 kg of the same age were selected out of the 60 birds and were randomly allocated to the following 3 treatments: no stunning (control); 40% CO_2_ + 21% O_2_ + 39%N_2_ (G40%); and 79% CO_2_ + 21% O_2_ (G79%). These parameters were based on our previous study showing that stunning broilers with low CO_2_ levels (30%, 40%) improved meat quality (meat lightness, tenderness and drip loss) at 24 h postmortem compared with high CO_2_ levels (50%, 60%) [5]; 79% was the highest CO_2_ level based on the same percentage of O_2_. The GS procedures and the GS system were set according to our previous study [5], with individual birds stunned for 90 s. The experiment was performed outdoors in the middle of November in Beijing, China, where the temperature was approximately 10 °C.

### Sampling

Exsanguination was performed without stunning (control) or immediately after stunning (treatments) by severing the jugular vein and carotid artery on one side of the neck. Bleeding was allowed for 3 min. Duplicate blood (3 mL × 2) samples were collected in sterile centrifuge tubes during the bleeding, incubated at room temperature for 3 h, and centrifuged at 1,800×*g* (4 °C) for 10 min. The supernatant was collected, mixed well, equally distributed into 6 sterile tubes (0.5 mL each), and stored at − 20 °C for the detection of serum cortisol, free triiodothyronine (FT3), free thyroxine (FT4), triiodothyronine (T3), and thyroxine (FT4). The ratio of T3 to T4 and the ratio of FT3 to FT4 were expressed as T3/T4 and FT3/FT4, respectively.

Carcass skinning and sampling were initiated 20 min after bleeding and concluded within 45 min postmortem. Meat samples were taken from the pectoralis major (representative of breast muscle) and musculus iliofibularis (representative of thigh muscle) for each meat variable. Muscles (approximately 0.2 g each) from the left side of the carcass were collected in duplicates and placed into 0.5 mL DNase /RNase free tubes, immediately placed in liquid nitrogen, and subsequently stored at − 80 °C until the analysis of gene expression. Five pieces of muscles (approximately 10 g each) were taken from the left side of the carcass. One sample was immediately stored at − 20 °C after sampling at 45 min postmortem (0 d at 4 °C), and the other 4 were stored at 4 °C for 1, 3, 6, and 9 d and then transferred to − 20 °C for preservation. One piece of muscle (10 g each) from the right side of the carcass was obtained and stored at 4 °C to measure the enzyme activity of total superoxide dismutase (SOD) and GST. Breast and thigh muscles (musculus iliofibularis and ventral muscles, 30 g each) from the right side of the carcass were also obtained and stored at 4 °C for 0, 1, 3, 6, and 9 d to measure the postmortem meat color. TBARS and enzymes were analyzed after storage at − 20 °C for 2 and 3 months, respectively.

### Determination of serum variables

Serum FT3, FT4, T3, T4 and cortisol were measured with commercial radioimmunoassay kits (Kit No. S10950167, S10950166, S10930047, S10930048, and 20150620, respectively) purchased from Biological Technology Research Institute of Northern China (Beijing, China). All four hormones were detected based on the principle of homogeneous competitive inhibition. The measurement processes for each hormone was similar. For example, for the evaluation of FT3, the competitor of FT3 was a free triiodothyronine derivative T3X. When the serum protein and antiserum were present at the same time, T3X could bind to the FT3 antibody but not to the serum protein. Samples (serum or distilled water, or different concentrations of standard products), ^125^I-labelled T3X, and a known amount of antibody of FT3 (primary antibody) were mixed together and incubated at 37 °C for 1 h. The serum FT3 in the samples (or standards) competed with ^125^I-labelled T3X for the limited binding sites of the FT3 antibody. Then, the donkey anti sheep immune separation agent (secondary antibody) was added, mixed well, placed for 15 min at room temperature, and centrifuged at 1,800×*g* for 15 min. The supernatant was discarded, and the sediment was subjected to radioactive count (cpm) with the radio-immunity analyzer (XH6080, Nuclear Instrument Factory, Xi’an, China). The linear standard curve can be obtained by calculating the binding ratio of standard samples (a serious of concentrations). According to the binding ratio of the serum, the content of serum FT3 can be calculated from the standard curve. The competitors for FT4, T3, T4, and cortisol were free thyroid hormone derivative ^125^I-T4X, ^125^I-T3, ^125^I-T4 and ^125^I-cortisol, respectively. The FT3, FT4, T3, T4 and cortisol were distinguished by each of their specific primary antibodies. Serum uric acid was measured with a uric acid kit (Nanjing Jiancheng Bioengineering Institute, Nanjing, China). Detection procedures were performed according to the manufacturer’s instructions.

### Determination of meat color

Meat color was measured in duplicate along the central axis of the meat surface using the CIE-Lab system with a Chroma Meter CR-300 (Minolta, Osaka, Japan), and the average value was counted as the final value. The values of lightness (L*), redness (a*), yellowness (b*) and total chromatic aberration (E*, total chromatic aberration, i.e. overall meat color) at 45 min, 0, 1, 3, 6, and 9 d postmortem were recorded.

### Determination of lipid peroxidation and antioxidant capacity

The TBARS were measured at 45 min, 1, 3, 6, and 9 d postmortem and expressed as malondialdehyde (MDA) as in our previous study [12]. The activity of the SOD enzyme at 45 min and 1 d postmortem, the activity of GST at 45 min, 1, and 3 d postmortem, and protein expression at 45 min, 1, 3, 6, and 9 d postmortem were analyzed with SOD, GST and protein (coomassie brilliant blue method) kits (Nanjing Jiancheng Bioengineering Institute, Nanjing, China), respectively, according to the manufacturer’s instructions.

### Determination of gene expression

The mRNA expression levels of *p38MAPK* (*p38*), extracellular-signal regulated kinase 2 (*ERK*2, also known as *MAPK1,* synonymous with *MAPK3* or *ERK1* in *Gallus gallus*), c-Jun N-terminal kinase 1 (*JNK1*), c-Jun N-terminal kinase 2 (*JNK2*), *Nrf2*, the alpha3, kappa 1, mu 2, and theta 1 isozymes of glutathione S-transferase (*GSTA3*, *GSTK1*, *GSTM2*, *GSTT1* respectively), Cu/Zn-superoxide dismutase (*SOD1*, or *Cu/Zn-SOD*), and Mn-superoxide dismutase (*SOD2* or *Mn-SOD*) were determined via real-time quantitative RT-PCR with beta-actin (*β-actin*) as an internal control. The stability of *β-actin* expression was tested through the statistical analysis of its CT values via one-way ANOVA with SPSS statistical software (Version 17.0, SPSS Inc., Chicago, IL). The CT values among treatments was not significantly different (*P* > 0.10) for both breast and thigh muscles, indicating that the mRNA expression of *β-actin* was stable under the present experimental condition. Thus, *β-actin* was used as an internal control. The isolation and reverse transcription of total RNA, and the quantitative real-time RT-PCR procedures were performed according to the methods described by Zhang et al. [22] using the same reagents and equipments. Briefly, total RNA was extracted from an 80 mg sample of the pectoralis major or musculus iliofibularis with TRIzol Reagent (Invitrogen Gibco-BRL, Bethesda, MD, USA) according to the manufacturer’s protocol. The quality of total RNA was assessed from the A260 nm/A280 nm absorbance spectra (in 10 mmol/L Tris-HCl, pH 7.5), Agilent Bioanalyzer 2100 (Agilent technologies, Santa Clara, CA, US). The RNA concentration was estimated from the absorbance at 260 nm. The RNA with A260 nm/A280 nm values between 1.8 and 2.1, an RNA integrity number ≥ 7.0 were considered qualified and were used for the reverse transcription. Quantitative real-time reverse transcription-PCR (RT-PCR) was performed with the primers and PCR conditions used in our previous study [21]. The exception was that the primers and reaction conditions were the same between ERK2 in present study and ERK1/2 in previous study. Three pairs of primers were designed for each gene. The primer with the best amplification effect was chosen for the quantitative PCR. Before the detection of genes, the PCR assay was optimized by running serial annealing temperatures and dilutions of a template. The mRNA expression levels for each gene were normalized as the ratio to *β-actin* mRNA [21–23] in arbitrary units using the 2^−ΔΔCT^ method [24]. To note that, the stability of *β-actin *was not provided in references of [21–23], readers needed to do the stability confirmation of *β-actin* under experimental condition or use ideally 3 housekeeping genes if the methods in their paper were referred.

### Statistical analysis

All data were analyzed via one-way ANOVA with SPSS statistical software (Version 17.0, SPSS Inc., Chicago, IL) with each chicken as a replicate. Means were separated by Duncan’s multiple range tests. Relationships between each pair of variables were calculated with Bivariate Pearson’s correlation coefficients. The results are presented as the means and S.E.M. in the Tables. *P* ≤ 0.05 indicates a significant difference.

## Results and discussion

### Meat color

The effects of GS methods on the color of breast and thigh meat stored from 45 min to 9 d postmortem at 4 °C are shown in Table 1; however, data for which *P* was > 0.1 were omitted for conciseness. Compared with the control group, L*_1 d_ (*P =* 0.03) and L*_9 d_ (*P <* 0.01) in the breast meat were increased, whereas E*_1 d_ (*P =* 0.05) and E*_3 d_ (*P <* 0.01) in the breast meat and b*_3 d_, b*_6 d_ and E*_6 d_ in the thigh meat were decreased in both the G40% and G79% groups. The L*, a*, b* and E* values reflected the lightness, redness, yellowness and the overall meat color of the broilers. This finding indicated that gas stunning with CO_2_ decreased the meat color quality of both breast (1, 3 and 9 d postmortem) and thigh (3 and 6 d postmortem) meat regardless of whether a low or high concentration of CO_2_ was used for stunning. In addition, values of a*_1 d_ (*P =* 0.06), E*_6 d_ (*P =* 0.04) and E*_9 d_ (*P =* 0.02) in the breast meat and a*_6 d_ and b*_1 d_ in the thigh meat (*P <* 0.01) were the lowest in group G79%. Taken together, the present data suggested that GS with high stunning intensity (79% CO_2_) resulted in the worst quality of meat color in both breast and thigh meat after storage from 1 to 9 d postmortem at 4 °C. Few reports have described the effect of CO_2_ stunning on meat color after long-term storage in poultry. The L* value is usually used as a symbol to estimate the incidence PSE condition in broiler breast meat [7]. Van Laack et al. [25] reported that breasts looked normal with L* values of 55 and those that looked pale had L* values of 60. In the present study, meat L* values indicated more paleness in gas stunned broilers compared with the control group. These values were below 59 in broilers stunned with both low CO_2_ and high CO_2_ concentrations. In our previous study, the lightness of breast meat at 24 h was increased by a GS of 50% CO_2_ compared with 30 ~ 40% CO_2_ [5]. However, lightness was not different in breast meat samples of the G40% and G79% groups in the present study. In a study by Kang and Sams [6], CO_2_ stunning (40 to 60% CO_2_) was associated with an increase in the L* value between 1.25 and 24 h postmortem and resulted in a greater L* value at 24 h postmortem compared to high-level CO_2_ killing (air displaced by CO_2_) in breast fillets from broilers. In a study from Battula et al. [7], the L* values were lower in the vacuum-stunning treatments at 0.75 and 4 h postmortem when compared with electrically stunned birds deboned at the same time. Birds subjected to Halal slaughter had more redness than birds that were exposed to stun-to-kill under a modified atmosphere containing a mixture of gases (40% CO_2_, 30% O_2_, and 30% N_2_) at 4 and 24 h postmortem [4]. The control group was slaughtered in a way very similar to the Halal slaughter method (without stunning, and transverse neck cut by severing the esophagus, trachea, jugular veins and carotid arteries) as described by Salwani et al. [4]. And the redness in breast muscle was also slightly higher in birds of the control group than the G40% group at 24 h postmortem. In all, both GS groups, especially G79%, decreased the meat color quality of both breast and thigh meat during long term storage at 4 °C.

**Table 1:** Effects of gas stunning methods on meat color in broilers

Color^2^	Storage time	Stunning methods^1^			*P*-value
		Control	G40%	G79%	
Breast meat					
L*	d 1	53.0 ± 0.81 ^a^	55.7 ± 0.79 ^b^	56.0 ± 0.77 ^b^	0.03
	d 9	52.7 ± 0.61 ^a^	56.0 ± 1.38 ^b^	58.2 ± 0.51 ^b^	< 0.01
a*	d 1	7.03 ± 0.41 ^b^	5.20 ± 0.72 ^ab^	4.48 ± 0.91 ^a^	0.06
b*	d 6	14.0 ± 0.46 ^b^	12.5 ± 0.76^ab^	11.6 ± 0.82 ^a^	0.08
E*	d 1	44.8 ± 0.68 ^c^	43.4 ± 1.05 ^ab^	41.7 ± 0.58 ^a^	0.05
	d 3	47.11 ± 0.41^b^	44.8 ± 0.68 ^a^	44.3 ± 0.48 ^a^	< 0.01
	d 6	43.1 ± 1.05 ^b^	40.9 ± 1.02 ^ab^	39.0 ± 0.90 ^a^	0.04
	d 9	44.6 ± 0.53 ^b^	41.9 ± 1.00 ^b^	40.7 ± 1.05 ^a^	0.02
Thigh meat					
a*	d 6	7.12 ± 0.58 ^b^	7.79 ± 0.92 ^b^	4.16 ± 0.20 ^a^	< 0.01
b*	d 1	12.9 ± 0.63 ^a^	15.8 ± 0.97 ^b^	10.7 ± 0.55 ^a^	< 0.01
	d 3	15.5 ± 0.38 ^b^	11.5 ± 1.46 ^a^	11.2 ± 0.89 ^a^	0.01
	d 6	13.7 ± 1.78 ^b^	6.00 ± 0.59 ^a^	7.73 ± 0.77^a^	< 0.01
	d 9	8.66 ± 2.20 ^a^	15.2 ± 2.06 ^b^	12.7 ± 1.21 ^ab^	0.08
E*	d 6	45.0 ± 1.02 ^b^	40.7 ± 1.19 ^a^	40.1 ± 0.89 ^a^	< 0.01

^1^Control = without stunning; G40% = 40% CO_2_ + 21% O_2_ + 39%N_2_; G79% = 79% CO_2_ + 21% O_2_

^2^L* = lightness; a* = redness; b* = yellowness; E* = total chromatic aberration. Color was measured for meat that was stored at 4 °C for 0, 1, 3, 6 and 9 d post-mortem. However, data with *P* > 0.1 were omitted in the table

^a–c^ Means with no common superscripts within a row differ significantly (*P* ≤ 0.05) or exhibit differences that approach significance (0.05 < *P* < 0.10); *n* = 6; values are expressed as the means ± S.E.M

### Lipid peroxidation

**Fig. 1:**
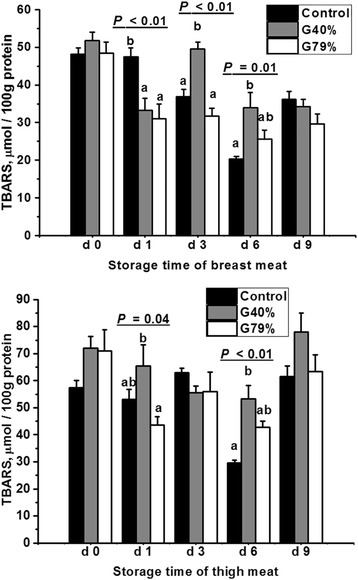
Effects of gas stunning methods on TBARS in skeletal muscles of broilers. TBARS is expressed as μmol malondialdehyde/100 g protein in meat, and meat was stored at 4 °C. Control = without stunning; G40% = 40% CO_2_ + 21% O_2_ + 39%N_2_; G79% = 79% CO_2_ + 21% O_2_. ^a–b^ Means with no common superscripts within a row differ significantly (*P* ≤ 0.05), *n* = 6

The level of lipid peroxidation was represented as TBARS (or MDA) in this study. The effects of GS methods on muscle TBARS are presented in Fig. 1. The TBARS levels at 1 d (*P* < 0.01) were decreased in breast muscle in both GS groups compared with the control group; TBARS levels at 3 d (*P* < 0.01) and 6 d (*P* = 0.01) postmortem in breast muscle were increased after GS with 40% CO_2_ compared to GS with 79% CO_2_. The highest levels of TBARS were observed in the breast meat in the G40% group at 3 and 6 d postmortem (*P* < 0.01) and in the thigh meat at 1 d (*P* = 0.04) and 6 d (*P* < 0.01). Consistent with the present study, TBARS_45 min_ and TBARS_24 h_ production in either breast meat or hind meat was not significantly different between broilers stunned with a CO_2_ of 40 and 60% in our previous study [12]. However, few reports have described the effect of CO_2_ stunning on TBARS in poultry after longer term storage. The data of present study showed that lipid peroxidation could be exacerbated in skeletal muscles during long-term storage at 4 °C in broilers stunned with 40% CO_2_ compared with broilers stunned with a high concentration (79%) of CO_2_ or slaughtered without stunning.

### Relationship between lipid peroxidation and meat color

The “free” iron in haemoglobin/myoglobin can affect meat color through self-oxidation [11] and can facilitate lipid oxidation to cause meat to change color [8]. The red color of muscle is primarily due to the presence of oxymyoglobin, and the oxidation of heme iron from the ferrous to the ferric state produces a brownish color [13]. Additionally, a higher haemoglobin content in the meat from residual blood can lead to higher lipid peroxidation in the raw meat of broilers [10]. In the present study, the highest TBARS levels were associated with the lowest level of lightness, the highest level of redness and the highest overall meat color in breast muscle at 1 d postmortem. This finding indicated that lipid peroxidation affected by GS of 40% or 79% CO_2_ was moderate and was not harmful to the meat color. However, the reaction trend of TBARS was not consistent with most of the trends of meat color variables, indicating that lipid peroxidation was not the primary or direct reason for differences in meat color after CO_2_ stunning. Similarly, lipid peroxidation of breast meat was observed to not be the primary or direct reason for differences in meat color after electrical stunning in our previous study of electrical stunning [21]. Protein oxidation [26] and protein phosphorylation [27] are other important factors that affect meat’s sensory quality, and myoglobin oxidation as a part of protein oxidation could lead to meat discoloration [8]. Oxymyoglobin stability is affected not only by lipid peroxidation but also by the autoxidation of myoglobin and the oxidation of ferrous ions and ascorbic acid [13]. In all, lipid peroxidation was moderate and did not affect the meat color directly after CO_2_ stunning in the present study. Whether protein oxidation or phosphorylation affect meat color after GS requires further investigation.

### Serum variables

The effects of stunning methods on the serum variables in broilers are shown in Table 2. The up-regulation of cortisol aids protein hydrolysis for energy production through gluconeogenesis under stressful conditions [28] to help animals cope with adverse stresses such as increased ambient temperature, starvation, or long periods of transportation [29]. Cortisol is also negatively correlated with meat pH [30]. In the present study, serum cortisol was increased in group G40% compared with the control group and G79% group (*P* < 0.01), indicating that broilers stunned with a low CO_2_ concentration may have experienced the highest stress compared with the other treatments.

**Table 2:** Effect of gas stunning methods on serum variables in broilers

Serum variables^2^	Stunning methods^1^			*P*-value
	Control	G40%	G79%	
Cortisol, ng/mL	2.21 ± 0.23 ^a^	3.72 ± 0.33 ^b^	2.33 ± 0.21 ^a^	< 0.01
Uric acid, mg/mL	205 ± 17.8 ^a^	304 ± 26.2 ^b^	298 ± 13.1 ^b^	< 0.01
FT3, fmol/mL	2.29 ± 0.40	1.24 ± 0.14	1.82 ± 0.44	0.14
FT4, fmol/mL	3.14 ± 0.50 ^a^	5.21 ± 0.79 ^b^	3.74 ± 0.41 ^ab^	0.07
T3, ng/mL	1.11 ± 0.08 ^b^	0.67 ± 0.03 ^a^	0.85 ± 0.06 ^ab^	0.03
T4, ng/mL	38.5 ± 2.03	41.4 ± 4.00	41.2 ± 4.43	0.51
T3/T4, %	2.87 ± 0.20 ^b^	1.56 ± 0.12 ^a^	2.26 ± 0.25 ^ab^	0.06
FT3/FT4, %	77.3 ± 7.4 ^c^	24.7 ± 2.73 ^a^	49.3 ± 4.8 ^b^	< 0.01

^1^Control = without stunning; G40% = 40% CO_2_ + 21% O_2_ + 39%N_2_; G79% = 79% CO_2_ + 21% O_2_

^2^FT3, FT4, T3, T4 represent free triiodothyronine, free thyroxine, triiodothyronine, and thyroxine, respectively

^a–b^Means with no common superscripts within a row differ significantly (*P* ≤ 0.05) or exhibit differences that approach significance (0.05 < *P* < 0.10); *n* = 6; values are expressed as the means ± S.E.M

Uric acid is an important non-enzyme antioxidant that is probably oxidized by ROS to allantoin during acute oxidative stress [31]. In the present study, serum uric acid was increased in both of the GS groups (*P* < 0.01) compared with the control group, opposing to the changing trend of TBARS_1 d_. This finding indicated that broilers stunned with both low and high concentrations of CO_2_ activated the non-enzyme system to protect muscle from further oxidative injury at the initial stage within 1 d postmortem of meat storage. As opposed to the GS group, serum uric acid was increased in the ES group that received the lowest current and a high frequency compared with the birds that had not been stunned [21, 32] and the ES group that received the highest current and lowest frequency [21].

T3 is one type of thyroid hormone, and most T3 is produced outside the thyroid from T4 deiodination. Most T3 and T4 are bound to binding proteins in the serum. FT3 and FT4 are the active states of the thyroid hormones, and FT3 has a stronger physiological effect than FT4 [33]. In the present study, serum T3 (*P* = 0.03) and FT3/FT4 (*P* < 0.01) levels were decreased, T3/T4 tended to be lower (*P* = 0.06), and FT4 (*P* = 0.07) tended to be higher in the G40% group compared with the control group. This finding suggested that GS with 40% CO_2_ depressed the conversion from T4 to T3, resulting in lower circular T3 in the serum of broilers compared with broilers slaughtered without stunning. The reduced peripheral conversion of T4 to T3 in the presence of normal thyroid hormone secretion is a characteristic of Low-T3 syndrome, which occurs in a variety of nonthyroidal illnesses [34]. The decrease in plasma T3 concentration rather than T4 concentration was related to heat production [35]. Therefore, the decrease in serum T3, T3/T4 and FT3/FT4 also indicated that the body tried to reduce heat production so that energy could be spared to manage stress in G40% in broilers. As opposed to GS, both high-current, low-frequency ES and low-current, high-frequency ES depressed the conversion from T4 to T3 [21].

### Relationship between lipid peroxidation and serum variables

The lowest levels of T3 and FT3/FT4 and the highest levels of cortisol were present in the G40% group, indicating that birds stunned with a low concentration (40%) of CO_2_ may have experienced the highest stress and needed more energy to cope with the stress of GS. However, a non-enzyme antioxidative system (e.g., uric acid) was activated to manage the challenge of oxidative stress in the skeletal muscle of broilers stunned with both types of GS. Oxidative stress was induced in broilers with glucocorticoid (corticosterone) treatment [36]. The increase in TBARS at 3 and 6 d postmortem in meat was consistent with the increase in cortisol (a stress hormone, another glucocorticoid) in the serum, suggesting that GS with 40% CO_2_ induced the highest degree of oxidative stress in skeletal muscles.

Thyroid hormones play particularly important roles in maintaining antioxidant balance, since both hyperthyroidism and hypothyroidism have been observed to be associated with oxidative stress in animals and humans [34]. In the current study, serum T3 was negatively correlated with TBARS_6 d_ both in the breast and thigh meat (*r* = − 0.63, *P* < 0.01 and *r* = − 0.47, *P* = 0.05, respectively). T3/T4 was negatively correlated with TBARS_6 d_ in the breast meat and TBARS_6 d_ in thigh meat (*r* = − 0.57, *P* = 0.01; and *r* = − 0.53, *P* = 0.03, respectively). FT4 was positively correlated with TBARS_6 d_ in the breast meat and nearly correlated with TBARS_1 d_ in the thigh meat (*r* = 0.57, *P* = 0.01; *r* = 0.42, *P* = 0.07, respectively). Similarly, the increased levels of TBARS_1d_ and TBARS_9d_ in breast meat were consistent with higher serum levels of FT3 and FT4 in the ES group that received 86 mA and 1,000 Hz compared to the group that received 130 mA and 60 Hz [21]. However, serum T3 was slightly positively correlated to the TBARS_1d_ in breast meat in our previous study of ES [21]. At the tissue level, hypothyroidism reinforces oxidative stress, which in turn worsens hypothyroidism by inhibiting deiodinases, thus establishing a vicious circle [34]. Therefore, data from the present study indicated that the decrease in serum T3, T3/T4 and the increase in FT4 may reinforce oxidative stress, leading to aggravated lipid peroxidation at 6 d postmortem in meat from broilers stunned by GS with 40% CO_2_. It would be interesting to further study the mechanism of how thyroxine affects lipid peroxidation in the meat from broilers stunned by CO_2_.

### Relationship between lipid peroxidation and the activity of antioxidant-related enzymes

In the present study, *GST* was chosen as a representative phase II enzyme that can be regulated by MAPK/Nrf2/ARE antioxidant signaling pathway, whereas SOD was chosen as a representative common antioxidant enzyme. The effects of stunning methods on the activity of these enzymes in skeletal muscles of broilers are displayed in Table 3. The activity of SOD_1__d_ was highest in both breast (*P* < 0.01) and thigh muscles (*P* = 0.02), whereas the activity of GST_45 min_ in the breast (*P* = 0.04) and GST_1 d_ (*P* < 0.01) in the thigh muscle was the lowest in broilers stunned with 79% CO_2_. This finding suggested that both the phase II enzyme and antioxidant enzymes in the skeletal muscles were affected by GS methods. However, the pattern of GST activity and of SOD activity was not consistent with the pattern of TBARS levels in corresponding breast or thigh muscles. This finding suggested that changes in TBARS might result from the joint action of both phase II enzymes and antioxidant enzymes. Another possible explanation is that the up-regulation of antioxidative status in the muscle by preslaughter stress could not counteract the subsequent development of accelerated lipid oxidation in meat [37]. Further efforts are needed to determine whether other phase II enzymes and antioxidant enzymes participate in the antioxidant process in skeletal muscle under different GS situations.

**Table 3:** Effects of gas stunning methods on antioxidant-related enzyme activity in skeletal muscles of broilers

Items^2^	Stunning methods^1^			*P*-value
	Control	G40%	G79%	
Breast muscle				
SOD_45 min_	13.6 ± 2.07	20.6 ± 2.49	15.4 ± 1.86	0.10
SOD_1 d_	29.5 ± 1.48^a^	26.2 ± 0.87^a^	39.8 ± 4.03 ^b^	< 0.01
GST_45 min_	5.56 ± 0.34^b^	5.65 ± 0.37^b^	4.28 ± 0.41 ^a^	0.04
GST _1 d_	2.77 ± 0.45	2.41 ± 0.26	3.13 ± 0.24	0.34
GST _3 d_	1.89 ± 0.29	1.71 ± 0.40	1.53 ± 0.47	0.82
Thigh muscle				
SOD_45 min_	15.9 ± 0.73	17.1 ± 2.39	23.1 ± 3.26	0.10
SOD_1 d_	33.9 ± 1.73^a^	36.4 ± 1.56 ^ab^	44.2 ± 3.29 ^b^	0.02
GST_45min_	5.17 ± 0.44	6.49 ± 1.19	5.64 ± 0.22	0.76
GST _1 d_	4.81 ± 1.15 ^a^	5.73 ± 1.19 ^b^	4.69 ± 0.22 ^a^	< 0.01
GST _3 d_	2.01 ± 0.37	1.11 ± 0.34	2.30 ± 0.48	0.12

^1^Control = without stunning; G40% = 40% CO_2_ + 21% O_2_ + 39%N_2_; G79% = 79% CO_2_ + 21% O_2_

^2^SOD and GST represent total superoxide dismutase and glutathione S-transferase, respectively. Subscripts represent storage times

^a–b^Means with no common superscripts within a row differ significantly (*P* ≤ 0.05); n = 6; values are expressed as the means ± S.E.M

### Gene expression in the MAPK/Nrf2 signaling pathway

**Fig. 2:**
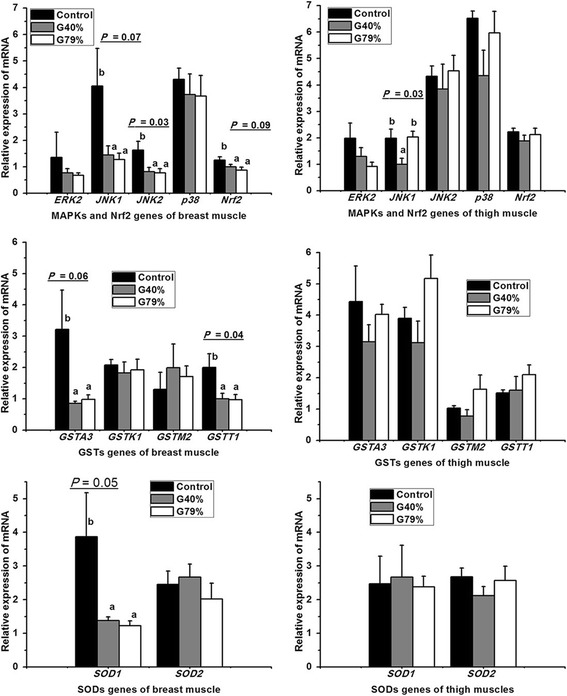
Effects of gas stunning methods on mRNA levels of MAPK/Nrf2/ARE signalling pathway molecules and SODs in the skeletal muscles of broilers. *MAPK* = mitogen-activated protein kinase; *Nrf2* = nuclear factor-erythroid 2-related factor 2, or chicken erythroid-derived CNC-homology factor, or *ECH*; ARE = antioxidant responsive element; SODs = superoxide dismutases. *ERK2* = extracellular-signal regulated kinase 2 (*ERK*2, also known as *MAPK1,* synonymous with *MAPK3* or *ERK1* in *Gallus gallus)*; *JNK1* and *JNK2* represent c-Jun N-terminal kinase 1 and 2, respectively. *GSTA3*, *GSTK1*, *GSTM2*, and *GSTT1* represent the alpha3, kappa 1, mu 2, and theta 1 isozymes of glutathione S-transferase, respectively; *SOD1* = Cu/Zn-superoxide dismutase; *SOD2* = Mn-superoxide dismutase. Genes of the thigh muscle with *P* > 0.1 were omitted from the table. Control = without stunning; G40% = 40% CO_2_ + 21% O_2_ + 39%N_2_; G79% = 79% CO_2_ + 21% O_2_. ^a–b^ Means with no common superscripts within a row differ significantly (*P* ≤ 0.05) or exhibit differences approaching significance (0.05 < *P* < 0.10), *n* = 6

The mRNA expression of *MAPK*s (*ERK*2, *JNK1*, *JNK2*, *p38*) and *Nrf2* in skeletal muscle is presented in Fig. 2. *MAPK*s can mediate transcription factor activity by phosphorylation [19]. When the nuclear transcription factor *Nrf2* is phosphorylated and activated by *ERK* and *p38*, it can be translocated to the nucleus, leading to the up-regulation of phase II enzyme expression (e.g., *GSTP1*) [38, 39] or the expression of genes encoding critical components of the glutathione (GSH)-dependent and thioredoxin-dependent antioxidant systems, along with those for the regeneration of NADPH [40] to protect against oxidative injury. The mRNA level of *JNK2* decreased (*P* = 0.03), and the mRNA level of *JNK1* (*P* = 0.07) and *Nrf2* (*P* = 0.09) decreased slightly in breast muscle from both the G40% and G79% groups compared with the control group. This finding indicated that GS with both high and low CO_2_ concentrations potentially decreased the ability of the muscle to fight against oxidative stress and lipid peroxidation in the breast muscle. Birds in the control group were slaughtered without stunning. Thus, they reacted to the neck cut with different degrees of struggling and flapping. This reaction may have caused the high variability of *JNK1* levels in the breast muscle of the control group. As opposed to GS, most of the genes in the MAPK/Nrf2/GST pathway of breast muscle were transcriptionally activated by low-current, high-frequency ES at 65 V and 86 mA compared with high-current, low-frequency ES (150 V and 130 mA) [21]. However, among the genes in the pathway, only *JNK1* was affected by GS in the thigh muscle; *JNK1* expression in the thigh muscle was lower in the G40% group compared with the control and G79% groups (*P* = 0.03). This finding demonstrated that among the several MAPK pathways, only *JNK1* and *JNK2* were sensitive to GS stress in both breast and thigh skeletal muscles. This observation was consistent with the report that in general, the *p38* and *JNK* pathways typically respond to various extracellular stress signals, whereas the ERK pathway responds to growth factor signals [19]. The different levels of gene expression between the breast and thigh may be due to the presence of different types of fibers in these muscles, as observed in our previous study [41, 42]. The present study also demonstrated that GS with both low and high CO_2_ concentrations slightly suppressed the MAPK/Nrf2 signaling pathway in the breast muscle of broilers compared with broilers that not been stunned.

### Gene expression of phase II enzymes

The phase II enzymes (such as GST) that are mediated by *Nrf2* contribute to cytoprotection and potentially to the self-repair of cells exposed to oxidative stress [38]. Both cytosolic (particularly the *GST*A, *GST*M, and *GSTT* gene families) and microsomal *GST* catalyse the conjugation of reduced GSH with a wide variety of electrophiles produced from oxidative stress, including oxidized DNA and lipid [43]. The mRNA levels of the *GST* isozymes in the skeletal muscles are presented in Fig. 2. The transcription of *GSTT1* (*P* = 0.04) was down-regulated, and the transcription of *GSTA3* was slightly lower (*P* = 0.06) in the breast muscle of both the G40% and G79% groups compared with the control group. The current data indicated that GS of both low and high CO_2_ concentrations slightly suppressed the transcription of *GST* isozymes in the breast muscle of broilers compared to broilers that had not been stunned. The *cis*-acting element ARE which can be found in the *GST* and other genes, is responsive to ROS and may allow eukaryotic cells to sense and respond to oxidative stress [14]. *MAPK* can modulate antioxidant enzyme expression through the Nrf2/ARE axis [16], leading to the expression of phase II antioxidant enzyme in various cell types [17, 18]. Consistent with the down-regulation of *MAPK*s (*JNK2* and *JNK1*) and *Nrf2* mRNA expression, the decrease in *GST* (*GSTTA3* and *GSTT1*) gene expression indicated that the MAPK/Nrf2/ARE antioxidant signaling pathway was partially suppressed in the breast muscle of broilers stunned by both low and high concentrations of CO_2_. This again indicated that GS with both high and low CO_2_ concentrations potentially reduced the ability of the tissue to fight against oxidative stress and lipid peroxidation by suppressing the MAPK/Nrf2/ARE antioxidant signaling pathway in the breast muscle.

However, among the 10 genes in MAPK/Nrf2/ARE pathway of thigh muscles, only *JNK1* was significantly affected by GS methods, suggesting that this antioxidant signaling pathway in the thigh muscle is not sensitive to GS.

### Gene expression of *SOD*s

The SODs are a ubiquitous family of enzymes that efficiently catalyse the dismutation of superoxide anions [44] and eliminate excess ROS [45]. The mRNA levels of *SOD*s were examined in the present study to determine whether other antioxidant enzymes, in addition to the phase II enzymes (represented by *GST*s in the present study), were involved in the antioxidant process in the breast muscle of broilers stunned by different methods. In the present study, the mRNA expression of *SOD2* was not affected, while *SOD1* expression in the breast muscle was slightly suppressed (*P* = 0.05) by both of the GS treatments compared to the control group (Fig. 2). This finding indicated that antioxidant enzymes in addition to the phase II enzyme were also affected by GS and might be involved in the antioxidant process in the breast meat of broilers. Additionally, the struggling and flapping associated with slaughter varied in birds in the control group, which might be the reason for a high level of variability in the *SOD1* level in the breast muscle of the control group.

### Relationships among the MAPK/Nrf2/ARE pathway and antioxidant enzymes, lipid peroxidation and meat color

Although the MAPK/Nrf2/ARE antioxidant pathway was partially transcriptionally suppressed in the breast muscle by GS methods, the enzyme activities of SOD within 1 d and of GST within 3 d postmortem were affected differently compared to the genes, and the changing pattern of TBARS did not correlate with gene expression or enzyme activity. This finding indicated that regardless of the CO_2_ concentration, GS suppressed the MAPK/Nrf2/ARE signaling pathway regulated antioxidant system and SOD1 in breast muscle at a transcriptional level. However, the signal transduction from mRNA to the enzyme activity was interrupted, leading to accelerated lipid oxidation in the breast meat of broilers stunned with 40% CO_2_. Similarly, signal transduction from the mRNA of these genes to enzyme activity was interrupted postmortem under the condition of electrical stunning stress [21]. This interruption might be due to changes in the intracellular and extracellular environment (e.g., pH, stop of blood supply) in the dead cells of the slaughtered birds. The production of ROS in the thigh muscle was lower in birds stunned with 40% CO_2_ at 45 min postmortem in our previous study [12]. This decrease in ROS may have suppressed the inner antioxidant systems, leading to the highest lipid peroxide levels in the G40% group in the present study. However, further studies are needed to clarify mRNA expression in the signaling pathway and the mechanisms that interrupted signal transduction.

Under the condition of oxidative stress, the phase II enzymes contribute to cytoprotection and potentially to the self-repair of cells [38], and the joint regulation by *Nrf2* of enzymes and transporters involved in detoxification and enzymes involving in glutathione metabolism ensures that these pathways are co-induced [40]. In the present study, the gas stunning stress partially suppressed the MAPK/Nrf2/ARE antioxidant signaling pathway at a transcriptional level, resulting in a potentially higher risk of lipid peroxidation in the breast muscle of CO_2_-stunned broilers. Additionally, oxidative stress (higher serum cortisol and FT4, and lower T3 and T3/T4) was observed only in the G40% group, leading to the highest level of lipid peroxidation in both breast and thigh meat from broilers stunned with 40% CO_2_.

## Conclusion

The variations in meat color quality and lipid oxidation after GS stunning were similar in the breast and thigh muscles, whereas the expression levels of the MAPK/Nrf2/ARE antioxidant signaling pathway genes were different between the two types of skeletal muscles. The present study reveals that stunning stress, oxidative stress and lipid peroxidation were exacerbated in the meat of broilers stunned with 40% CO_2_ versus those stunned with 79% CO_2_ and the control group, whereas meat color quality and the transcription of MAPK/Nrf2/ARE signaling pathway genes in the breast muscle was partially decreased by gas stunning irrespective of CO_2_ concentration. Lipid peroxidation was not the primary or direct reason for changes in meat color after gas stunning. The antioxidant status was not able to overcome the lipid peroxidation induced by a low concentration of CO_2_. Future investigations of protein expression and phosphorylation are needed to further study the mechanism of how signal transduction in this pathway, from mRNA expression to enzyme activity, was interrupted in the skeletal muscles of CO_2_-stunned broilers.

## Abbreviations

ES: Electrical stunning
GS: Gas stunning
MAPK: Mitogen-activated protein kinase
Nrf2: Nuclear factor-erythroid 2-related factor 2, or chicken erythroid-derived CNC-homology factor, or ECH
p38: p38MAPK
ARE: Antioxidant responsive element
ERK: Extracellular-signal regulated kinase
JNK1: c-Jun N-terminal kinase 1
JNK2: c-Jun N-terminal kinase 2
SOD1: Cu/Zn-superoxide dismutase
SOD2: Mn-superoxide dismutase
GSTA3, GSTK1, GSTM2, GSTT1: Represent the alpha 3, kappa 1, mu 2, and theta 1 isozymes of glutathione S-transferase, respectively
Control: Without stunning
G40%: 40% CO_2_ + 21% O_2_ + 39%N_2_
G79%: 79% CO_2_ + 21% O_2_
FT3: Free triiodothyronine
FT4: Free thyronine
T3: Triiodothyronine
T4: Thyroxine
L*: Lightness
a*: Redness
b*: Yellowness
E*: Total chromatic aberration
